# Visual narrative comprehension: Universal or not?

**DOI:** 10.3758/s13423-019-01670-1

**Published:** 2019-12-09

**Authors:** Neil Cohn

**Affiliations:** Tilburg School of Humanities and Digital Sciences, Department of Communication and Cognition, Tilburg center for Cognition and Communication, Tilburg University, P.O. Box 90153, 5000 LE Tilburg, The Netherlands

**Keywords:** Visual narrative, Narrative, Comics, Temporal cognition, IQ test, Theory of mind, Picture arrangement

## Abstract

Visual narratives of sequential images – as found in comics, picture stories, and storyboards – are often thought to provide a fairly universal and transparent message that requires minimal learning to decode. This perceived transparency has led to frequent use of sequential images as experimental stimuli in the cognitive and psychological sciences to explore a wide range of topics. In addition, it underlines efforts to use visual narratives in science and health communication and as educational materials in both classroom settings and across developmental, clinical, and non-literate populations. Yet, combined with recent studies from the linguistic and cognitive sciences, decades of research suggest that visual narratives involve greater complexity and decoding than widely assumed. This review synthesizes observations from cross-cultural and developmental research on the comprehension and creation of visual narrative sequences, as well as findings from clinical psychology (e.g., autism, developmental language disorder, aphasia). Altogether, this work suggests that understanding the visual languages found in comics and visual narratives requires a fluency that is contingent on exposure and practice with a graphic system.

## Introduction

Sequential images are so pervasive in contemporary society that we may take their understanding for granted. Comics or instruction manuals are generally assumed to be simple and universally comprehended, underlying their use in intelligence testing and clinical assessments (Kaufman & Lichtenberger, 2006; Wechsler, 1981), and as stimuli in a wide range of anthropological and experimental research. A presumed accessibility has placed visual narratives in practical contexts like instructions (Martin & Smith-Jackson, 2008; Spinillo & Dyson, 2001) and humanitarian aid materials (Fussell & Haaland, 1978; Stenchly, Feldt, Weiss, Andriamparany, & Buerkert, 2019), and has underscored efforts advocating for using comics in education (Cary, 2004; Sousanis, 2015) and science and health communication (Farinella, 2018; M. J. Green & Myers, 2010). Despite these widespread assumptions, a wealth of research suggests that sequential images are not simplistic or universally transparent, and may require a *fluency****–*** i.e., *a proficiency acquired through exposure to and practice with* a system of visual narrative. Such fluency is argued as comparable to the natural, extensive, and, often passive, exposure and practice required to comprehend language.

This review synthesizes research on *sequential* image understanding. *Sequential images* broadly are juxtaposed images bound by meaningful connections, including instruction manuals and signage. *Visual narratives* are a type of sequential images, often drawn, which convey a continuous event sequence, typically to tell a story, as in comics and picture stories. First, we examine why visual narratives may be perceived to be universal, and explore their structure and processing. Next, we address their fluency in cross-cultural, developmental, and clinical contexts. Finally, we analyze the implications of these findings, not the least being the necessity of understanding an overlooked, yet fundamental and ubiquitous mode of human expression.

## Reasoning for universality

There are several reasons sequential images might be presumed to be understood universally. *Single pictures* are often iconic ***–*** they resemble their meaning (e.g., Peirce, 1931) ***–*** and naïve beliefs about drawing hold that they represent what is seen by vision or a mental image, not culturally constrained and learned schematic patterns (like language). If drawings represent what people see, they should be universal, since all people ostensibly have the same perceptual capacities. Differences in producing drawings thus reduce to “talent,” despite the assumed universality in their understanding (for review, see Cohn, 2014a; Willats, 2005; Wilson, 1988).

Similar assumptions extend to *sequential* images: if event understanding is universal, and images simply depict perception, sequential images depicting events should also be transparent. Researchers have thus assumed that static, drawn sequential images provide a transparent way to study action planning (Tinaz, Schendan, Schon, & Stern, 2006; Tinaz, Schendan, & Stern, 2008), theory of mind (Baron-Cohen, Leslie, & Frith, 1986; Sivaratnam, Cornish, Gray, Howlin, & Rinehart, 2012), social intelligence (Campbell & McCord, 1996), sequential reasoning (Zampini et al., 2017), temporal cognition (Boroditsky, Gaby, & Levinson, 2008), and discourse comprehension (Gernsbacher, Varner, & Faust, 1990), among other cognitive abilities.

Beliefs about the simplicity and universality of sequential images are no doubt reinforced by their ubiquity. Sequential images extend back to cave paintings, and appear in many historically and culturally diverse contexts (McCloud, 1993; Petersen, 2011). In contemporary societies, sophisticated visual narratives appear in comics, picture books, and storyboarding, and sequential images appear in instruction manuals and signage. This ubiquity seems to have no specific origin ***–*** i.e., visual narratives were not “invented” in one place and then spread across the world. Rather, creating sequences of graphic images appears to be a “universal” potential of human communication and cognition.

Despite this ubiquity as a “universal” aspect of human communication, it does not mean that visual narratives are *universally* understood. In line with *Visual Language Theory* (VLT), we argue that creating and understanding sequential images is analogous to language (Cohn, 2013b). Though language is a cognitively “universal” and “innate” system in that all typically developing human brains have the cognitive structures necessary to speak or sign languages (Jackendoff, 2002), language *fluency* is not developmentally inevitable and requires exposure to and practice with an external system. For spoken or signed languages, only in unfortunate circumstances do individuals not receive this requisite experience (Goldin-Meadow, 2003). For visual narratives, a lack of drawing skill may be more widespread and culturally permissible, since they are less integrated into everyday interactive communication (cf. Wilkins, 1997/2016). However, this does not exclude visual narrative fluency from the same interaction between Nature and Nurture as fluency in language, despite different cultural assumptions and practices.

## The structure of visual narratives

**Fig. 1:**
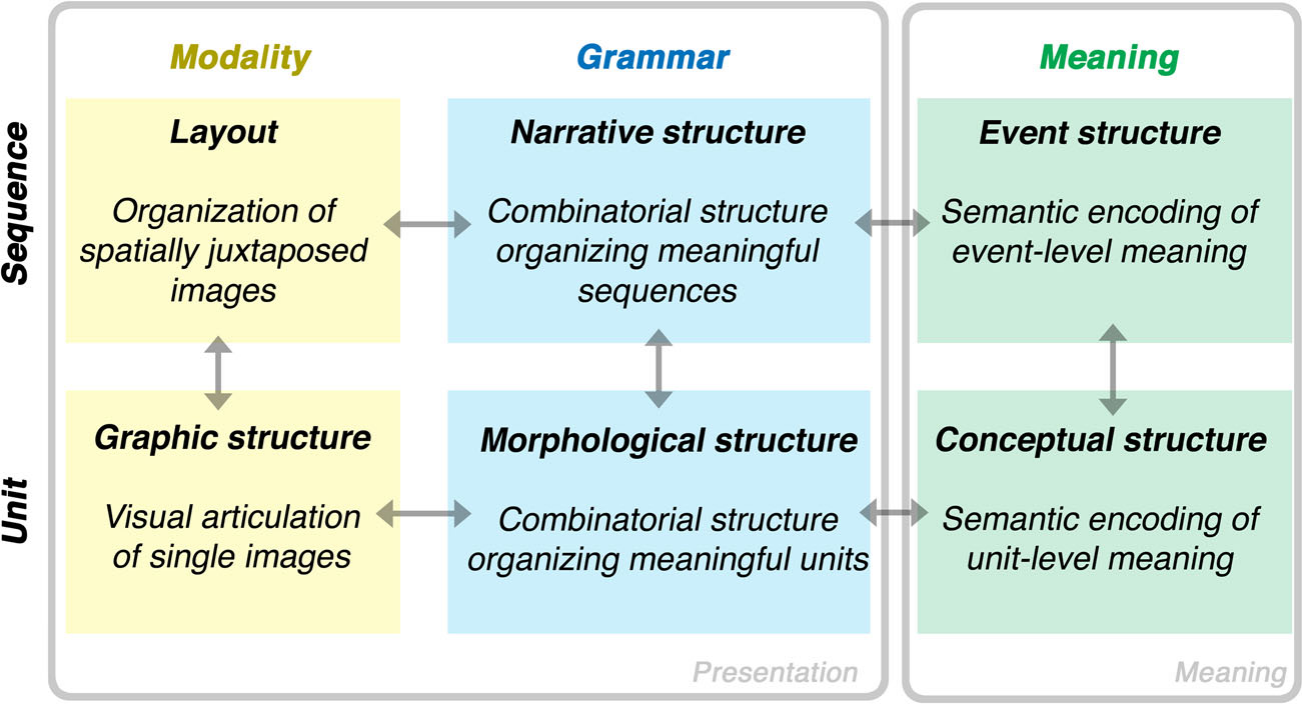
A model of the architecture of visual narratives dividing across the primary components of a modality, grammar, and meaning for both the unit and sequence levels

Before exploring the fluency of comprehending visual narratives, we first must address their structure and processing. The “visual language” used in drawn narratives involves an interaction between three primary structures, similar to the parallel architecture of linguistic systems (Jackendoff, 2002): A *meaning* expressed by a *modality* (here: visual-graphic marks), which is organized using combinatorial *grammatical* structures. As depicted in Fig. 1, these structures operate across both units (here, individual images) and sequences of those units.

*Graphic structures* govern the lines and shapes that make up a visual depiction, analogous to phonological structures organizing the modality of sound in spoken languages. This visual information maps to meanings, which a *morphological structure* organizes using the schematic patterns underlying basic drawing and mark-making (Willats, 2005; Wilson & Wilson, 1977) and combinatorial meanings in speech balloons, motion lines, and other symbols (Cohn, 2013b; McCloud, 1993). These graphic schemas constitute a “visual lexicon” stored in long-term memory, which combine to form novel drawings. Acquisition of these schemas characterizes “learning to draw” (Cohn, 2012; Wilson & Wilson, 1977), facilitated by exposure to and imitation of the pictures in a learner’s environment (M. V. Cox, 1998; Wilson, 1988; Wilson & Wilson, 1977).

While their iconicity allows for more widespread comprehension ***–*** and the illusion of transparency ***–*** pictures require familiarity to be understood, from simple images (for review see Arbuckle, 2004; de Lange, 2000) to combinatorial morphology (Cohn, 2013b; Nakazawa, 2016). When shared as systematic representations across individuals of a population, they constitute lexicons of different “visual languages” bound to historical, cultural, and/or functional contexts. Though image-units can be complex and modulated by proficiency, here we are concerned with *sequences* ordering these units, the structure of which is described in the top row of Fig. 1.

**Fig. 2:**
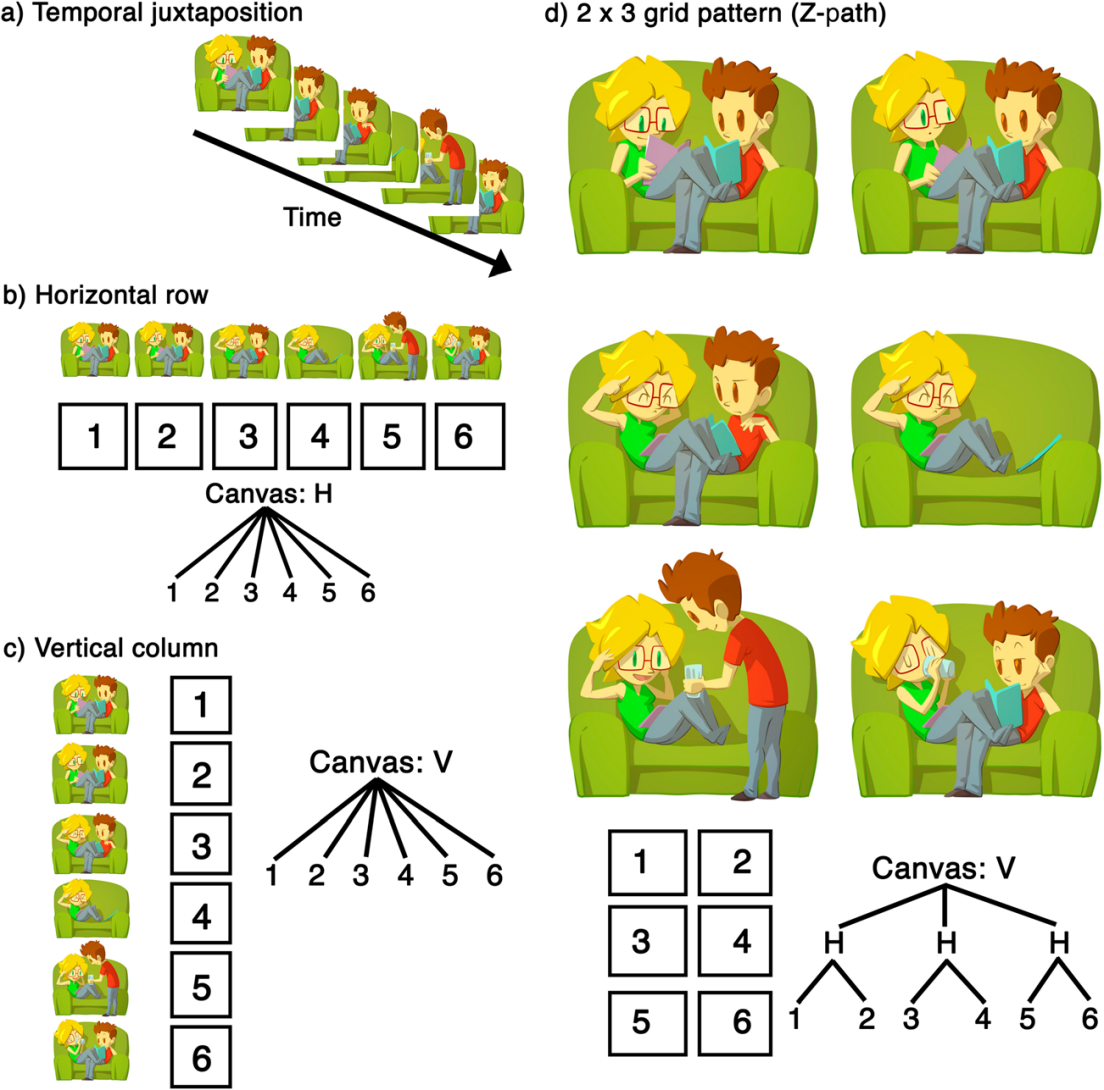
Variation in layout for *JA!* by Ángela Cuéllar and Jonás Aguilar (© 2016). A sequence could be conveyed one image at a time in a temporal sequence (**a**), or in spatial layouts of (**b**) a single vertical (V) column or (**c**) a single horizontal (H) row, or in (**d**) its original grid layout of three horizontal (H) rows each with two panels, embedded in a vertical (V) column

Sequential image-units ***–*** or *panels****–*** can manifest in different physical *layouts*, as in Fig. 2. *Temporally sequential* juxtapositions present images one after the other in *time*, as in Fig. 2a, be it unfurling in a slide-show, sketched on a chalkboard, or drawn in sand (Cohn, 2013b). *Spatially sequential* juxtapositions arrange images next to each other, whether one image per page (as in picture books) or linear horizontal (Fig. 2b) or vertical sequences (Fig. 2c). More complicated multi-panel layouts appear in comic pages, often expanding from a basic grid pattern (Fig. 2d). Given a constant reading order, the same meaningful content can have various arrangements (horizontally, vertically, grids, etc.). Thus, layout is separate from, yet interfaces with, structures governing meaning (Cohn, 2013b). While most readers follow ordering principles from writing systems, e.g., the left-to-right-and-down “Z-path” or its reverse (Cohn, 2013a; Spinillo & Dyson, 2001), complex layouts may require alternate routes constrained by conventionalized navigational principles (Cohn, 2013a).

Beyond physical juxtaposition, sequential images also involve meaningful connections, and possibly use a narrative structure for ordering, framing, and modifying that content (Cohn, 2013b). Simple sequences require only semantic relationships. *Unordered* sequences are visual lists in instruction manuals and signage (e.g., image sequences with icons indicating “no dogs, no skateboarding, no smoking” etc.). Simple *ordered* sequences are basic stepwise instructions (i.e., step 1, 2, 3 … ), but complex sequences use embedding and narrative categories.

**Fig. 3:**
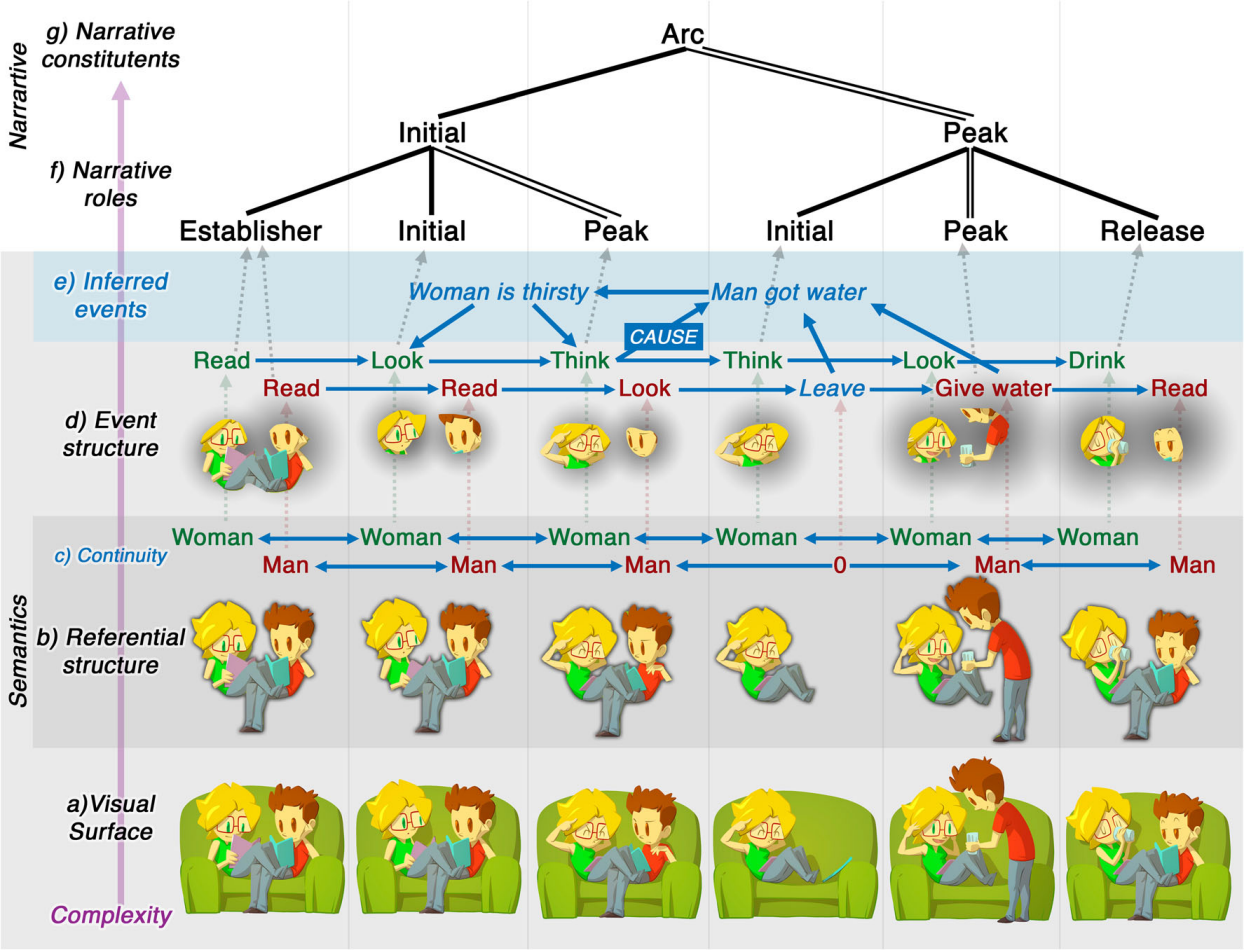
Depiction of the semantic and narrative content for a comic from *JA!* by Ángela Cuéllar and Jonás Aguilar (© 2016)

Ordered visual sequences involve several levels of structure, as in Fig. 3. This sequence depicts a man and woman sitting on a couch reading. The woman then thinks very hard, and the man subsequently gets up and brings her a glass of water. The inference is that she was thirsty, and her intense thinking commanded him to bring her water non-verbally.

A comprehender must first access the basic semantic information in each image. In Fig. 3b, the images referentially depict a man, a woman, a couch, books, and a glass of water. The depictions also show events (Fig. 3d): both woman and/or man are sitting, she dramatically thinks (panels 2 and 3), he hands a glass of water (panel 5), and she drinks (panel 6). This information remains in the image units, which each frame the whole characters. Despite having no overt boundaries (i.e., a drawn frame), we assume they are six discrete units.

To construe these units as a sequence, a comprehender must track components across images and observe their changes. First, elements in one image must be recognized as the same referential entities in subsequent images (Bornens, 1990; Saraceni, 2001; Stoermer, 2009). Thus, a *continuity constraint* guides the understanding that each image does not depict different characters, but repeats the *same characters* across images. Continuity requires mapping visual features to a common referential entity. In Fig. 3c, the same woman and man are interpreted in all panels cued by the same hair, clothing, etc. If their shirts or hairstyles changed between images, it would challenge the continuity of these same characters repeating across frames.

Nevertheless, some changes in depiction motivate sequential meaning. Thus, an *activity constraint* characterizes that, despite continuity, visual changes might cue shifts in time, viewpoint, or causation. In Fig. 3, the woman’s postural change between panel 1 (book up, head down) and panel 2 (book down, head up) signals that she raised her head. Because not all repetitions nor changes in elements signal successive states, a comprehender must distinguish cues for continuity and activity from irrelevant alterations (e.g., changes in size, occlusion, etc.).

Without these constraints sequential images could not be recognized *as a sequence*. A lack of referential continuity would render each entity in a panel as a unique character, not the same character repeated (i.e., in Fig. 3 each panel shows different men and women). Continuity without activity would imply the same character in unrelated scenes (i.e., each panel shows the same man and woman, but in unconnected scenes). Finally, absence of both continuity and activity would render each image as separate characters in unconnected scenes. These constraints only arise in spatially sequential juxtapositions that require repetition across spatial arrangements. Temporally sequential juxtapositions need no such constraints, as long as elements in a single space persist in time (Cohn, 2013b). Thus, layout can have consequences for interpretation.

When comprehenders recognize continuity across panels, they can then be sensitive to changes across those images, and this knowledge is incorporated into a growing mental model of the scene (Cohn & Kutas, 2015; Loschky, Hutson, Smith, Smith, & Magliano, 2018). Shifts between images may be construed as changes between characters, spatial locations, time, and/or events (McCloud, 1993; Saraceni, 2001), consistent with changes across units in verbal or filmed discourse (Loschky et al., 2018; Magliano, Higgs, & Clinton, 2019). Incremental changes may only require mappings into a mental model, but larger discontinuity may prompt inferences, requiring greater updating (Cohn & Kutas, 2015; Loschky et al., 2018). In Fig. 3, the minimal changes between panels 1***–***3 would require little updating, but panels 4 and 5 demand inference (why did the man disappear and reappear?). These inferences relate to the referential structure (the man didn’t vanish in panel 4), the event structure (his absence in panel 4 is because he got a glass of water), and/or the intentions and goals of characters (the woman is inferred as being thirsty in panel 2, which can only be realized by panel 5).

Simple sequences require only basic connections, with each image holding the same status (as in visual lists). More complicated sequencing may differentiate the roles images play, create hierarchic segments, connect panels across distances, and/or negotiate ambiguities with multiple interpretations. Such characteristics require a *narrative structure*, which organizes semantic information (Cohn, 2013b), as in Fig. 3f. Sequences may introduce characters (Establisher), before starting actions and events (Initial) which eventually climax (Peak) and resolve (Release). Though image content can cue such categories, ultimately narrative structure is separate from meaning (for review, see Cohn, 2019b). Narrative roles also apply at structurally higher levels (Fig. 3g), where the first three panels set up (Initial) the climactic (Peak) final three panels (Cohn, 2013b; Cohn, Jackendoff, Holcomb, & Kuperberg, 2014). Further modification introduces complexity through repetition of narrative categories, zooms of information, and other constructional patterns (Cohn, 2013b, 2019a; Cohn & Kutas, 2017).

## Visual narrative processing

An emerging literature has begun examining how visual narratives are processed (Cohn, 2019b). This work primarily uses wordless visual sequences, and has implied connections between visual narrative and linguistic processing, implicating domain-general mechanisms (Cohn, 2013b; Magliano et al., 2019). Behavioral methods suggest that language and visual narratives share resources for inference generation (Magliano, Larson, Higgs, & Loschky, 2015) and segmentation (Magliano, Kopp, McNerney, Radvansky, & Zacks, 2012). Neurocognitive research has also implicated similar brain areas across verbal and visual narratives (Gernsbacher & Robertson, 2004; Robertson, 2000), including Broca’s and Wernicke’s areas (Cohn & Maher, 2015; Nagai, Endo, & Takatsune, 2007; Osaka, Yaoi, Minamoto, & Osaka, 2014).

Studies of event-related potentials (ERPs) implicate similar neural responses to semantic processing across domains in the “N400” ERP component (Kutas & Federmeier, 2011). Though first observed to unexpected words in sentences (Kutas & Hillyard, 1980), N400 effects also appear to anomalous and/or unexpected information in visual narratives (for review, see Cohn, 2019b). This semantic information integrates into a growing mental model, which updates with situational changes (characters, events, etc.). Such updating is indexed by a “P600” (Brouwer, Crocker, Venhuizen, & Hoeks, 2016; Kuperberg, 2013) and thereby is involved in the continuity and activity constraints: Larger P600s appear to both congruous and incongruous changes of characters across images (Cohn & Kutas, 2015, 2017), consistent with P600s appearing to referential discontinuity in language (van Berkum, Koornneef, Otten, & Nieuwland, 2007).

Overlapping neurocognitive mechanisms are also implied between combinatorial structures (narrative, syntax). Violations of syntactic structure in sentences have elicited (left) anterior negativities, associated with combinatorial processing, and P600s, associated with updating as a structural revision (Hagoort, 2017). Similar ERP components are evoked in visual narratives by violations of constituent structures and narrative patterns (for review, see Cohn, 2019b). The similarities between ERPs to language and visual narratives parallel observations of shared mechanisms between language and music (Patel, 2003), despite involving different representations (words, images, notes).

Overall, these findings have several implications: First, visual narrative processing engages several interacting neurocognitive mechanisms across meaning (e.g., N400) and combinatorial (narrative) structure (e.g., anterior negativities, P600). Second, these cognitive resources may overlap with those implicated for sequencing in language and music. Third, insofar as they may be domain-general and multifaceted, these mechanisms are not strictly associated with perception or event cognition. Fourth, even if visual narratives use domain-general processes, fluency in the graphic modality is still required to access them. This too is presumably similar to language: though the linguistic system accesses domain-general mechanisms, fluency in specific languages ***–*** whether spoken or signed ***–*** are required to elicit such processing, and later acquisition or lack of fluency can be consequential on its development (Goldin-Meadow, 2003).

## Experimental methods using visual narrative

Before turning to visual narrative fluency, we must first describe methods and tasks using wordless visual narratives that inform these findings. As in Table 1, these tasks often balance several characteristics. Only sometimes are such tests administered to test visual narrative fluency directly, with most used to test other aspects of cognition. Contemporary research on visual narratives uses additional measures such as segmentation, response times, brainwaves, and other psycholinguistic methods.

**Table 1:** Tasks involving visual narratives used in the psychological sciences. See text for references using each task

Tasks	Arrangement	Narration	Inference	Questions
Picture arrangement task (PAT)	x			
Temporal card arrangement task (TCAT)	x			
Narration elicitation task (NET)		x		
Sequential reasoning task (SRT)			x	
Fill in the blank task (FITBT)			x	
Sequence completion task (SCT)			x	
Narrative comprehension task (NCT)				x

The most common method is the *picture arrangement task* (PAT), which asks participants to arrange several images into a coherent sequence. Answers are scored relative to a “correct” target sequence. This task appears across many disciplines, including in general intelligence (IQ) tests (WAIS-IQ, WISC) and clinical assessments (Kaufman & Lichtenberger, 2006; Wechsler, 1981). A PAT variant is the *temporal card arrangement task* (TCAT), which takes the spatial layout of the arrangement ***–*** i.e., in a horizontal row, vertical column, circular shape, etc. ***–*** to indicate spatial metaphors for time (Boroditsky et al., 2008).

Other tasks ask participants to *infer* information about a sequence. *Fill-in-the-blank tasks* (FITBTs) ask participants to guess the content of a missing panel (Nakazawa & Nakazawa, 1993a), while *sequence completion tasks* (SCTs) or *sequential reasoning tasks* (SRTs), ask participants to place an image at the start, middle, or end of a picture sequence (A. L. Brown & French, 1976; Zampini et al., 2017). The *Comic Strip Task* uses the same technique intending to assess theory of mind (Sivaratnam et al., 2012). Exposure to visual narratives precedes some of these tasks, often as PATs, introducing a *recall* component to the completion task.

Many fields use *narrative elicitation tasks* (NETs), where participants describe the story in an image sequence (sometimes preceded by a PAT). These tasks include the Frog Stories (Berman & Slobin, 1994), Jackal and Crow (Carroll, Kelly, & Gawne, 2011), Family Problems (Carroll, Evans, Hoenigman, & San Roque, 2009; San Roque et al., 2012), Circle of Dirt (Eisenbeiss, McGregor, & Schmidt, 1999), the Edmonton Narrative Norms Instrument (Schneider, Hayward, & Dubé, 2006), and others. The elicited narrations are then analyzed for various linguistic and/or cognitive properties.

Finally, *narrative comprehension tasks* (NCTs) present participants with visual narratives, followed by a comprehension period with a battery of questions (e.g., detail questions, inferential questions, recall, recognition, etc.).

## Cross-cultural sequential image comprehension and production

Despite the assumptions of universality, people from certain cultural backgrounds may not spontaneously construe images *as sequential*. Here, we review findings for both comprehension and production.

### Cross-cultural visual narrative comprehension

The non-universality of sequential images began emerging in cross-cultural contexts as researchers sought to use wordless sequential images for non-verbal communication, often motivated by practical, humanitarian, or educational efforts. Nevertheless, various populations did not construe their expected meanings. The consistent finding was an inability to recognize the *continuity constraint*, with each image instead interpreted as an isolated scene.

For example, researchers in Nepal sought to use wordless picture sequences to convey information about nutrition, hygiene, and environmental concerns (Fussell & Haaland, 1978). However, less than 50% of respondents understood the left-to-right ordering, many did not understand continuity in a three-panel sequence, and only 3% recognized that an image pair compared events. Similarly, respondents in Papua New Guinea had difficulty construing sequences (A. Bishop, 1977) and/or temporal orders (Cook, 1980), though familiarity with comics improved a sequential interpretation (Cook, 1980). Comparable findings have been observed in elicitation tasks, where Awiakay speakers from Papua New Guinea interpreted each image as its own story, and similar findings are reported about Aboriginal Australian Umpila speakers (San Roque et al., 2012, p. 153).

Several studies from Africa report similar findings. An older study in Kenya found that respondents had trouble construing sequences, but were somewhat better at construing pairs of images (Holmes, 1963). Yet, referential continuity was hard to construe even in pairs of “before-after” frames for Bantu (Zulu and Tsonga) workers in South Africa (Winter, 1963). Several studies have found continuity interpreted less often for native Africans compared to their European counterparts, including for Bantu populations (Duncan, Gourlay, & Hudson, 1973), native South Africans (Liddell, 1996, 1997), with the Basotho people (Jenkins, 1978), and in Botswana (Byram & Garforth, 1980), with results modulated by age, acculturation, literacy, and exposure to graphics. Lower proficiency on the PAT was observed for young men from the Ganda tribe (Uganda), despite proficient verbal (English) and math skills (John McFie, 1961), and for illiterate compared to literate Sudanese participants, while 80% of participants “failed to respond adequately” to the PAT in a pilot study (Khaleefa & Ashria, 1995).

Temporal card arrangement tasks (TCATs) yield similar mixed findings (Boroditsky et al., 2008). While industrialized participants use consistently sequenced layouts (Fuhrman & Boroditsky, 2010; Levinson & Majid, 2013; Spinillo & Dyson, 2001), more variable layouts were produced by native individuals in Australia (Gaby, 2012), South America (P. Brown, 2012; Le Guen & Pool Balam, 2012), and Papua New Guinea (Fedden & Boroditsky, 2012; Levinson & Majid, 2013), among others. Some Yucatec Mayan speakers even confounded the test itself, piling pictures vertically rather than into a spatially juxtaposed layout (Le Guen & Pool Balam, 2012). While few studies report on comprehension of the image sequences, participants’ literacy is said to influence their construal (Gaby, 2012; Le Guen & Pool Balam, 2012; Levinson & Majid, 2013).

Finally, unexpected construals of sequential images often go unreported. Rafael Núñez (p.c.) described fieldwork with Kensy Cooperrider with the Yupno of Papua New Guinea (e.g., Núñez, Cooperrider, Doan, & Wassmann, 2012). They attempted to use a TCAT with images of a man’s beard getting longer, but respondents construed them as four different people (i.e., “brothers,” because they looked similar). Lauren Gawne (p.c.) reports similar challenges with NETs with Lamjung Yolmo speaking participants in Nepal. One respondent was “not literate in storybook conventions” (Gawne, 2016, p. 144) and first described the images with few sequential connections, and then interpreted juxtaposed panels as multiple birds, rather than a single bird across frames (Gawne, 2016, p. 144). This participant was older, and younger participants did not make similar construals since they learned “standard visual literacy” in schooling. These anecdotes are important because when respondents do not perform as expected, the result is perceived as a “failed” experiment and remains unreported in the scientific literature.

Overall, respondents who did not construe sequential images *as a sequence* come from rural communities with little or no exposure to visual narratives (i.e., comics or picture books). Such exposure, along with literacy, led to greater likelihood of sequential construal. Finally, several of these studies are old, and may not reflect such populations’ current understandings given possible adoption of Western culture (including comics). Nevertheless, the implications persist: not everyone comprehends sequences of images as sequential.

### Diversity in cross-cultural visual narrative systems

Just as languages differ around the world, visual narratives vary cross-culturally in their narrative patterns (Cohn, 2019a), and such differences modulate their readers’ comprehension (Cohn & Kutas, 2017). This diversity means that proficiency applies for visual sequences in general and for culture-specific patterns. This would be analogous to the distinction between language competency generally, and fluency in specific languages around the world. Thus, while lack of exposure may contribute towards non-sequential construals, another possibility is comic-like visual narratives may compete with indigenous systems.

For example, Aboriginal communities (Arrernte, Warlpiri) in Central Australia use narrative sand drawings that unfurl temporally in a single space (J. Green, 2014; Wilkins, 1997/2016). Wilkins (1997/2016) reports that Arrernte respondents had difficulty construing comic strips as sequential events, instead interpreting each image as its own scene. While such results could be interpreted as an inability to understand sequential images, Wilkins posits that the spatially sequential layout in comics conflicts with the Arrernte’s temporally sequential indigenous system. Such layout differences affect several levels of visual narratives’ structure, including continuity (Cohn, 2013b).

Several researchers stress a relationship between the layout of sequential images and literacy in a written language. Literacy correlates with left-to-right layouts in TCATs (Gaby, 2012; Le Guen & Pool Balam, 2012; Levinson & Majid, 2013), and basic sequential image comprehension (Fussell & Haaland, 1978). Literacy’s influence may not relate to content. Rather, navigating any spatial layout could be transferable across modalities. TCATs demonstrate this experimentally (Fuhrman & Boroditsky, 2010), but it also occurs conventionally: American comics follow the left-to-right order of English writing, while Japanese manga follow the right-to-left order of written Japanese. For respondents inexperienced with visual narratives, borrowing the linear navigation of writing for image sequences may bootstrap the recognition of *content* as continuous. (Alternatively, literacy accompanies acculturation to visual narratives, which may go unreported.) Thus, visual narrative fluency is not reliant on literacy, but literacy may help acquire fluency in *certain visual narrative systems* given the shared layout structures and ramifications of spatial juxtaposition on continuity.

### Cross-cultural visual narrative production

Additional insight comes from sequential image production. Brent and Marjorie Wilson spent decades researching children’s visual narratives across the world using a “Draw a Story” test which asked children to draw a narrative into six empty frames (see Wilson, 2016 for review). In the USA, Australia, Finland, and Egypt, middle-class urban children (ages 9 and 12 years) produced comparable structures and themes (Wilson, 2016), often imitative of comics (Wilson, 1974). In children’s drawings from Japan, where manga (“comics”) are immersive throughout culture, nearly all 6-year-olds could produce visual narratives, often with greater coherence and complexity than their counterparts around the world (Wilson, 1988). Nearly all of them imitated manga (Toku, 2001; Wilson, 1999, 2016; Wilson & Wilson, 1987).

Studies in Egypt well illustrate the effect of a lack of exposure (Wilson, 2016). In contrast to suburban Egyptian children (in Cairo), with exposure to illustrated books and comics, children from a rural village (Nahia), had little access to drawn visual culture, despite watching television, including American cartoons. Though they had verbal narration abilities, *only 4****–****8%* of these village 9-year-olds drew coherent visual narratives sequentially connecting contents of images (Wilson, 2016). They instead drew sequences of “frozen vignettes” ***–*** i.e., lacking continuity ***–*** with isolated objects or events (Wilson & Wilson, 1987). Around 50% of the older village children (12-year-olds) drew coherent sequences, but just used step-by-step linear sequencing without robust narrative structure (Wilson, 2016).

## Development of visual narratives

We now turn to a different aspect of fluency, with the developmental trajectory of sequential image comprehension and production. Here, sufficient exposure is assumed ***–*** i.e., with access to comics, cartoons, and picture books ***–*** unless specified otherwise.

### Development of sequential image comprehension

The developmental trajectory of sequential image understanding progresses incrementally (Bornens, 1990; Trabasso & Nickels, 1992; Trabasso & Stein, 1994). At early ages, children do not seem to comprehend *sequencing* of sequential images. Two-year-olds attend to narrated elements in picture stories read by parents, but with poor comprehension (Kaefer, Pinkham, & Neuman, 2017). Children at or below the age of 4 years do not construe characters repeated across images as the same entities (Bornens, 1990), do poorly with PATs (Friedman, 1990; Weist, Atanassova, Wysocka, & Pawlak, 1999; Weist, Lyytinen, Wysocka, & Atanassova, 1997), and show little ability to choose correct sequence endings (Zampini, Suttora, D'Odorico, & Zanchi, 2013; Zampini et al., 2017). When narrating picture stories, children up until around age 4 or 5 years perceive each image in a sequence as an isolated event: They typically describe the contents of each image, rather than integrate sequential information across images (Berman, 1988; Poulsen, Kintsch, Kintsch, & Premack, 1979; Trabasso & Nickels, 1992; Trabasso & Stein, 1994).

Nevertheless, children as young as 3 years can understand causal relations between images of drawn objects ***–*** such as *cup-hammer-broken cup****–*** when no continuity constraint is required (Gelman, Bullock, & Meck, 1980). Thus, young children may recognize causal events, but struggle with sequential referential continuity. Indeed, 2- to 3-year-olds recognize event sequencing earlier than indicated by sequential image comprehension (O'Connell & Gerard, 1985), and children as young as 3 years will describe dynamic events in their narratives (Berman & Slobin, 1994).

Following these stages of referential and event recognition, children begin to construe sequential continuity, which was argued by Piaget and colleagues (Krafft & Piaget, 1925; Margairaz & Piaget, 1925) as beginning around age 7 or 8 years. Subsequent work observed children around the age of 6 or 7 years could better follow continuity when the images retained consistent backgrounds (Schweitzer & Schnall, 1970). More contemporary research has observed that children at the age of 4 years begin to understand cross-panel continuity and activity cues, reaching full understanding between 5 and 6 years (Bornens, 1990). These ages align with the shift from children describing isolated image units to narrating sequential events (Berman, 1988; Karmiloff-Smith, 1985; Paris & Paris, 2003; Poulsen et al., 1979; Shapiro & Hudson, 1991; Trabasso & Nickels, 1992; Trabasso & Stein, 1994). Children between 4 and 6 years also increasingly select accurate sequence-ending panels (Zampini et al., 2017), and are moderately good at discerning the causes or consequences of a sequence’s main event (A. L. Brown & French, 1976). Ages 4***–***6 years also appear to be the lower end for proficiency in the PAT (Consortium, 2015; Fivush & Mandler, 1985; Kato, 2006). Four-year-olds can arrange a previously seen sequence from memory better than a random sequence (A. L. Brown & Murphy, 1975). Similarly, recall from picture stories improves from fairly low around the age of 4 years (Poulsen et al., 1979) to decent by the age of 6 and 7 years (A. L. Brown, 1975; Poulsen et al., 1979),

Around the age of 5 years, children begin to proficiently infer content omitted from a sequence (Schmidt & Paris, 1978; Shaklee, 1976; Zampini et al., 2013; Zampini et al., 2017). Inferencing improves in sequences maintaining continuity of characters across images (Kunen, Chabaud, & Dean, 1987), and is not predicted by general intelligence or cognitive flexibility (Zampini et al., 2013). Also, children between 4 and 6 years of age increasingly discern the primary story elements of a narrative picture sequence (Hayward, Schneider, & Gillam, 2009; Poulsen et al., 1979; Silva & Cain, 2017), and generally improve in retelling ability and narrative comprehension between 4 and 9 (Milch-Reich, Campbell, Pelham, Connelly, & Geva, 1999; Paris & Paris, 2001, 2003; Schneider et al., 2006). This comprehension varies little for children of different ethnic backgrounds speaking different languages, but who live in the same culture (Verhoeven & Vermeer, 2006).

Though sequencing ability appears to begin between the ages of 4 and 6 years, fluency continues developing with age. PAT performance improves into later ages (A. L. Brown, 1975), reaching peak accuracy by the low teens (Nakazawa, 2005, 2016), as does understanding of narrative coherence (Bingham, Rembold, & Yussen, 1986) recall (Milch-Reich et al., 1999; Nakazawa, 2016; Nakazawa & Nakazawa, 1993b) and both bridging inferencing of missing content (Nakazawa & Nakazawa, 1993a; Schmidt, Paris, & Stober, 1979) and predictive inferencing about future content (Pallenik, 1986; Paris & Paris, 2003). Frequency of comic reading experience may modulate these abilities into later life (Nakazawa, 1997, 2004).

**Fig. 4:**
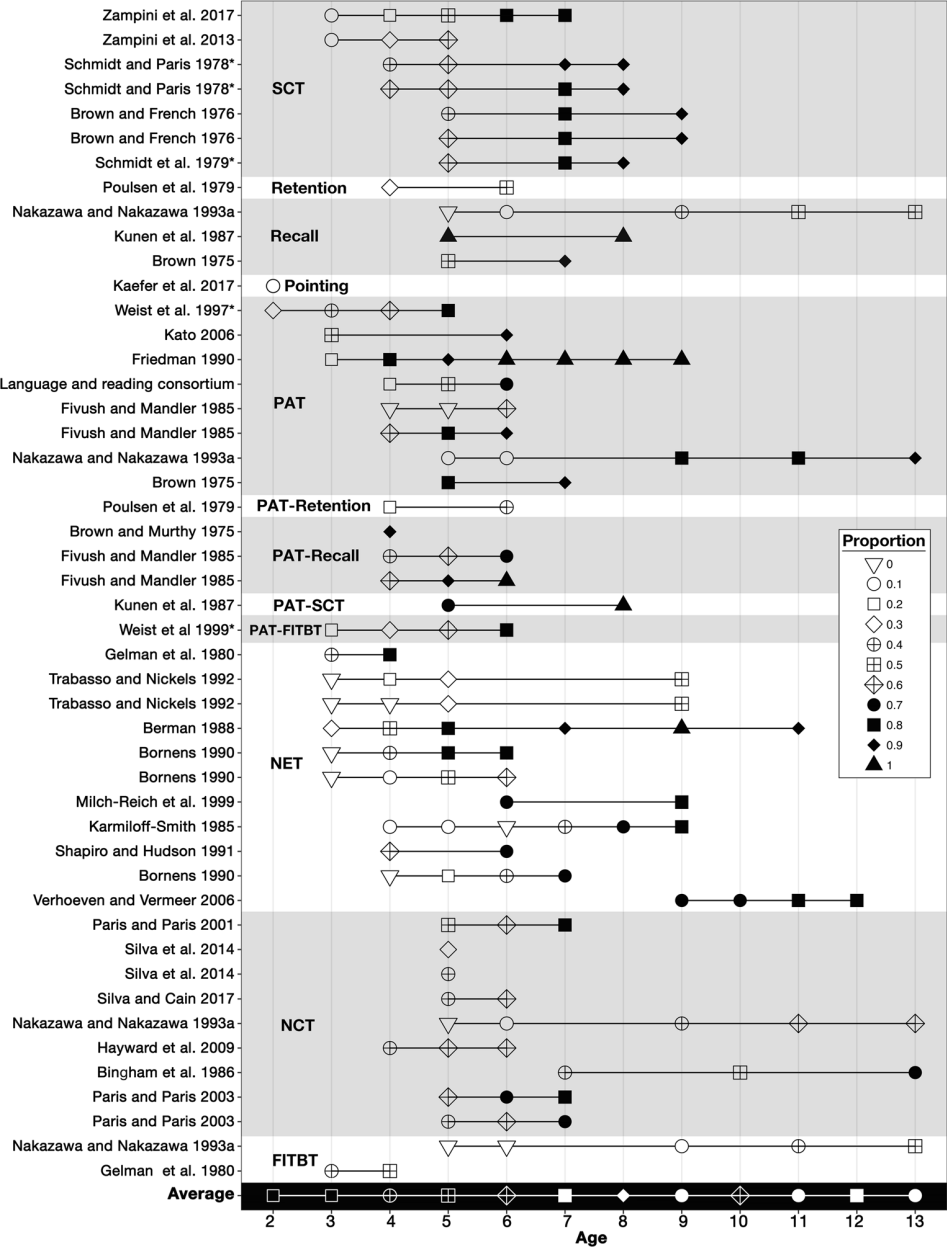
Age-related aspects of sequential image comprehension aggregated from developmental studies using visual narratives, normalized into proportions and rounded (indicated by markers). All scores report unmanipulated sequences types (not those using backwards, scrambled, or random sequences, etc.), and studies with mixed age groups here report the mean age per group (*). Grey and white bands and adjacent acronyms (see Table 1) depict different tasks assessed. Repeated entries index different sub-experiments

Figure 4 summarizes the results of several studies using visual narrative tasks. When possible, reported scores are provided, but proportions were calculated for those reporting only raw scores, which were divided by total possible correct (as stated in Results or Methods sections). For mixed age groups, the mean age per group is reported (references marked with *). Numbers are rounded where needed. Results show only comprehension of “ordered” or coherent narrative sequences, excluding manipulated sequences (scrambled, random, backward, etc.), and for neurotypical populations.

Despite the wide range of methods and researchers’ intents, consistent age-related effects can be observed. Overall, proficiency gradually rises from the age of 2 years through 8 years, with a crucial shift between 4 and 6 years of age. Average scores then decline, partially because studies adjust their complexity to the relative ages being tested. This trajectory is also striking because these studies claim to investigate diverse aspects of cognition ***–*** narrative comprehension, sequential reasoning, temporal cognition, causal inference, etc. ***–*** yet the age-related task results remain consistent. This suggests at least some role of fluency for the visual narratives used in the tasks themselves ***–*** an interpretation offered by few of the many studies analyzed.

This developmental trajectory may vary based on exposure and other social factors like socio-economic status. Bornens (1990) reports that less “culturally privileged” children recognized continuity later (5***–***7 years old) than other children (4***–***5 years old). Delayed development for less privileged children may be attributable to reduced exposure to visual narratives. Exposure may also explain varied proficiencies between children from different cultures (Weist et al., 1997), and why comics reading experience modulates proficiency even between college students (see below).

Finally, these abilities do not arise in isolation. During these ages, children develop many cognitive abilities potentially involved in sequential image understanding. For example, theory of mind develops before or during this time period (Wellman, Cross, & Watson, 2001). Certainly, the recognition of characters’ intentions and goals are involved in understanding stories, though probably not to assess basic referential continuity. Visual narrative development also coincides with verbal narrative abilities (Berman & Slobin, 1994; Trabasso & Nickels, 1992; Trabasso & Stein, 1994), which warrants more careful examination of both the amodal and modality-specific aspects of this development, which are often conflated.

### Development of sequential image production

The development of visual narrative production remains less clear. Comprehension and production skills may be asymmetrical ***–*** one may be able to read a comic, but not draw one (Stoermer, 2009). Visual narrative production must also be situated within the development of drawing more broadly. Children generally start with scribbling (1***–***3 years old) and using outlines to map drawn regions to conceptual volumes (3***–***8 years old), before eventually using lines to depict contours and edges (8***–***11 years old) (e.g., Willats, 2005). However, this developmental trajectory does not factor in cultural exposure to and practice with a graphic system (Cohn, 2012; M. V. Cox, 1998; Toku, 2001; Wilkins, 1997/2016; Wilson, 1988, 1999, 2016). Thus, proficiency in producing the graphic structure of drawings may develop concurrently with sequencing structures.

A developmental trajectory for basic drawing is important because unintelligible scribbles may still have “narrative” intent. Silver (2000) asked 3- and 5-year-olds to “retell” a videoed story through drawing. Though 3-year-olds prevalently produced scribbles, they still had intended meaning. Yet, in line with the trajectory for comprehension, 3-year-olds largely drew an inventory of characters, with few cues of time passing. In contrast, 5-year-olds depicted some temporality, juxtaposing images at a higher rate than 3-year-olds, though both produced short sequences (one to two panels), if at all.

Older groups also differ in their sequencing (Wilson, 2016). A study of 8-year-old Greek children found minimal sequencing when asking children to draw a story, instead finding individual images (Labitsi, 2007). Cox (1999) describes that in early stages of their narrative drawing exercises, 9- to 13-year-olds had “fairly primitive” cohesive devices, and captions were necessary to maintain a “continuous narrative.” An unpublished study by Durant (1981) found that 85% of children aged 11***–***13 years drew a story-prompt using a single frame, while adolescents aged 15***–***17 years chose equally between one-, four-, or six-panel sequences (cited in Smith, 1985).

The shift to sequencing pictures occurs between 5 and 7 years old (Wilson & Wilson, 1979a, 1982), and with exposure to comics, children can produce complex conventions like framing and narrative patterning (Wilson, 1974). Wilson and Wilson (1979b) found that 9- and 12-year-old American children changed the framing of a scene (e.g., full view to a close up) about once per a six-panel story, though 6-year-olds rarely did, a trait attributed to reading comics. Greater exposure to and practice with visual narratives leads to earlier proficiency: Nearly all 6-year-old Japanese children can produce coherent visual narratives, and complex framing changes are observed across most panels drawn by Japanese 12-year-olds (Wilson, 1988). Similar maturation in sequencing and layout has been observed from age 8***–***11 years for a Korean child (Kim, 2008).

Explicit instruction and guidance can enhance proficiency beyond passive exposure (Bitz, 2004a, 2004b). Stoermer (2009) found that 7- and 8-years-old students could develop complex stories with clear narrative arcs and developed backstories. Yet, they often had to be “coached image by image in order for the comic to be readable” (Stoermer, 2009, p. 191), particularly for correction of continuity errors across frames. Children may also abandon the stylistic details found in their individual images for more simplistic drawings in their sequential storytelling.

Sylvia Pantaleo has undertaken extensive instructional research with older children. She describes that 8- and 9-year-old children can adopt complex paneling that manipulates the size, shape, and/or semantics of panel frames, and even may play with meta-knowledge to narratively break their borders (Pantaleo, 2013b). Some children by the age of 12 years use sophisticated narrative modifiers with zoom panels and alternation patterns (Pantaleo, 2015), and conscious manipulation of framing, point-of-view (Pantaleo, 2012b, 2013a), and narrative “rhythm” (Pantaleo, 2019). They also may embed metafictional narration (Pantaleo, 2011, 2012a).

The developmental trajectory of visual narrative production remains understudied, with a wide range of variability in proficiencies observed across ages. Yet, this literature reinforces that proficiency is associated with exposure to visual narratives, either passive or instructed.

## Measurements of visual narrative expertise

The research above points towards the influence of exposure and practice with a system of visual narrative, particularly comics and picture books. Two threads of research have designed procedures for examining visual narrative proficiency. Such work implies that fluency may vary even amongst experienced readers of visual narratives.

### Chiba University Comic Comprehension Test (CCCT)

Since the early 1990s, psychologist Jun Nakazawa has examined manga comprehension and development. His battery of experiments includes recall and comprehension tasks, a PAT, a FITBT, and others, which comprise the *Chiba University Comic Comprehension Test* (CCCT) used to assess visual narrative proficiency (for review in English, see Nakazawa, 2005; Nakazawa, 2016). Nakazawa’s studies showed that visual narrative comprehension increases from childhood through adulthood (Nakazawa & Nakazawa, 1993a, 1993b). However, studies with adults (20 year-olds through to 60-year-olds) suggest that age alone does not modulate performance, and frequent manga readership both within and between age groups increased recall and comprehension (Nakazawa, 1997). Higher CCCT scores also appear for college students from Japan, where comic reading is ubiquitous, than the USA, where comic reading is less prevalent (Nakazawa & Shwalb, 2012). Similar differences arise between college students with or without experience reading manga in non-CCCT batteries (Lee & Armour, 2016). Nevertheless, the CCCT uses Japanese manga as materials, so these findings may assess manga comprehension specifically, rather than general fluency.

Finally, Nakazawa (2002) observed that an experienced manga reader had smoother eye-movements across panels of a page than a less-experienced reader, who focused more on the text than the images. Recent work has corroborated that inexperienced comic readers have more fixations across wordless comic pages than experienced readers, and inexperienced readers comprehended multimodal comics better than purely pictorial visual narratives (Zhao & Mahrt, 2018). Similarly, children fixate more and longer on panels in comics than children (Martín-Arnal, León, van den Broek, & Olmos, 2019). Altogether, this research further implies an interaction between age and experience on the comprehension of visual narratives.

### Visual Language Fluency Index (VLFI)

Recent research on visual narrative processing has assessed proficiency by measuring participants’ comic reading frequency. A *Visual Language Fluency Index* (VLFI; pronounced “vil-fee”) questionnaire asks participants’ to rate their frequency (on scale of 1 to 7) of reading comic books, comic strips, graphic novels, and Japanese manga, and of drawing comics, and their expertise (scale of 1 to 5) at comic reading and drawing. Ratings are given for both “currently” and “while growing up.” A *VLFI score* is then computed, weighing the metric more towards comprehension than production. VLFI scores are a standard protocol in “visual language” research^1^ (Cohn, 2013b).

VLFI scores correlate with many aspects of visual narrative processing, as in Table 2, such as ERP effects to image sequences (Cohn & Kutas, 2015; Cohn & Maher, 2015; Cohn, Paczynski, Jackendoff, Holcomb, & Kuperberg, 2012), response times to target images (Cohn et al., 2012), self-paced viewing times (Cohn & Maher, 2015; Cohn & Wittenberg, 2015), comprehension ratings (Cohn, Murthy, & Foulsham, 2016; Cohn & Wittenberg, 2015), accuracy judgements (Hagmann & Cohn, 2016), eye movements (Bateman, Beckmann, & Varela, 2018; Kirtley, Murray, Vaughan, & Tatler, 2018), and segmentation of narrative constituent structure (Cohn & Bender, 2017). Expertise also modulates participants’ preferences for reading order within comic page layouts (Cohn, 2013a; Cohn & Campbell, 2015)

**Table 2:** Studies on visual narrative processing reporting significant interactions between scores from the Visual Language Fluency Index questionnaire and behavioral or neurocognitive measures

Paper	Measure	Processing type	Effect of fluency
Cohn et al. (2012)	Reaction times	Narrative and semantic structure	Faster RTs for greater fluency
	ERP effects	Narrative structure	Larger amplitude ERP effects for greater fluency
Cohn & Kutas (2015)	ERP effects	Narrative and inference	Larger amplitude ERP effects for greater fluency
Cohn & Kutas (2017)	ERP effects	Narrative patterning	Different ERP components for familiarity with narrative pattern
Cohn & Maher (2015)	ERP effects	Morphological incongruity	Larger amplitude ERP effects for greater fluency
	Self-paced viewing times	Morphological incongruity	Longer viewing times to anomalies for greater fluency
Cohn & Wittenberg (2015)	Self-paced viewing times	Inference	Shorter viewing times for greater fluency
Cohn & Bender (2017)	Segmentation choices	Narrative segmentation	Segmentation choices were easier with greater fluency
Hagmann & Cohn (2016)	Accuracy	Narrative structure	Greater tolerance of incongruity for greater fluency
Cohn et al. (2016)	Ratings	Morphological familiarity and interpretations	Less tolerance of incongruity for greater fluency
Bateman et al. (2018)	Eye movements	Layout	More fluency associated with more consistent reading paths across panels
Kirtley et al. (2018)	Eye movements	Text-image relationships	Larger saccades within panels for greater fluency

Some research suggests that specific visual narrative patterns can modulate processing beyond a general “fluency.” An ERP study examined processing of a narrative pattern that appears more frequently in Japanese manga than in comics from the USA or Europe (Cohn, 2013b, 2019a). Across all participants, this narrative pattern evoked neural responses related to both combinatorial processing (anterior negativities) and mental model updating (P600) (Cohn & Kutas, 2017). A post hoc regression analysis with VLFI subscores then found that participants’ frequency of reading Japanese manga “while growing up” modulated these ERP effects. Findings with VLFI scores thus suggest that experience influences processing even between competent readers, both for visual narratives in general and for culturally specific patterns.

## Visual narrative in clinical populations

So far, this review suggests that typically developing humans understand and produce visual narratives when given exposure and practice. Might this fluency be disrupted in atypical conditions, such as in clinical populations? This question is especially important because visual narratives often appear in clinical tasks (for review, see Coderre, 2019). Many clinical populations struggle with the PAT and NET, beyond what is possible to review in this space. Here, we examine three populations that inform the relationship of visual narrative and linguistic processing: Autism Spectrum Disorder (ASD), Developmental Language Disorder (DLD), and aphasia.

### Autism Spectrum Disorder

Individuals with ASD have long been documented as struggling with language processing, yet have been said to do better with visual stimuli. Observations with visual narrative processing do not support such modality differences and imply more general processing challenges. Individuals with ASD are worse than neurotypical individuals in the PAT (Baron-Cohen et al., 1986; Johnels, Hagberg, Gillberg, & Miniscalco, 2013), and low PAT scores are consistent for individuals with ASD on IQ test batteries (Siegel, Minshew, & Goldstein, 1996). Lower PAT proficiency appears for children with ASD than adults (Siegel et al., 1996) and children with other language deficits (Allen, Lincoln, & Kaufman, 1991), which may relate to reading skills (Goldstein, Beers, Siegel, & Minshew, 2001). Individuals with ASD also have difficulty inferring missing content of an event sequence (Davis, Dautenhahn, Nehaniv, & Powell, 2007), and predicting the final image of a visual event sequence (Zalla et al., 2010). Children with ASD also provide shorter narrations of picture stories than typically developing children (Tager-Flusberg, 1995). Finally, an ERP study found that semantic processing (the N400) was attenuated for incongruities in both verbal and visual narratives for individuals with ASD compared to neurotypical controls (Coderre et al., 2018).

### Developmental Language Disorder

Connections across domains are also implied in studies with individuals with Developmental Language Disorder (DLD, previously known as Specific Language Impairment, or SLI), which is a diagnosis characterized by delayed language development but typical performance on non-verbal intelligence tests. DLD varies greatly across individuals, but problems acquiring syntax is a consistent feature (Leonard, 1998). Children with DLD do worse on the PAT than neurotypical controls, and this correlated with frontal brain regions analyzed with EEG (Nenadović, Stokić, Vuković, Đoković, & Subotić, 2014). Another study found children with DLD were proficient at non-verbal IQ tests, but had comparable deficits for answering questions about pictorial narratives as for verbal narratives (D. V. M. Bishop & Adams, 1992). Similar challenges occurred in the encoding and recall of image sequences beyond competencies with non-verbal IQ (D. V. M. Bishop & Donlan, 2005). Children with DLD also describe fewer aspects of picture stories than children with “pragmatic language impairment” (Botting, 2002) and typically developing children (Reilly, Losh, Bellugi, & Wulfeck, 2004), despite showing similar age-related improvements (Schneider et al., 2006).

### Aphasia

Studies of neurological damage caused by stroke or head injury are also informative, though not straightforward. The PAT is impaired by frontal lobe damage even in studies dating to the 1950s (e.g., J. McFie & Piercy, 1952), with right frontal lobe damage impairing more than left frontal damage (J. McFie & Thompson, 1972). However, PAT difficulty also occurs for patients with genetic mutations associated with left frontotemporal and anterior parietal lobe damage (De Renzi, Faglioni, Savoiardo, & Vignolo, 1966), with broad left (Fucetola, Connor, Strube, & Corbetta, 2009) and right hemisphere damage (Huber & Gleber, 1982; Marini, Carlomagno, Caltagirone, & Nocentini, 2005; Wallesch, Kornhuber, Köllner, Haas, & Hufnagl, 1983), and with Wernicke’s aphasia (Huber & Gleber, 1982). Recent fMRI studies with visual narratives implicate left hemisphere areas such as the basal ganglia and dorsolateral prefrontal cortex (Tinaz et al. 2006), and poor PAT scores appeared for a patient with a left basal ganglia lesion, who also had impaired language production abilities (Crescentini, Lunardelli, Mussoni, Zadini, & Shallice, 2008).

These mixed findings of impairments may relate to heterogenous aspects of visual narrative comprehension. Right and left hemispheric damage differently impair performance on the PAT, depending on the characteristics of the visual sequence (Veroff, 1978). Also, the choice of sequence endings was worse for right than left hemisphere damaged patients (Bihrle, Brownell, Powelson, & Gardner, 1986), but they differed in their errors: right hemisphere damaged patients chose structurally well-formed but incoherent endings, while left hemisphere damaged patients chose coherent but less structurally intact endings. Finally, recognition of congruity for a sequence-ending image was less accurate and took longer for a Wernicke’s aphasic than patients with conduction aphasia or anomia (Stead, Savage, & Buckingham, 2012).

Altogether, deficits in visual narrative comprehension vary across clinical populations, with implications for connections to language processing. Indeed, visual narratives are a complex system, and deficits may be heterogeneous across populations. Such complexity underscores the necessity of dedicated research on the structure and fluency of visual narratives. Given the growing sophistication of methods of studying the cognition of visual narratives using behavioral (reaction times, self-paced viewing times) and/or neurocognitive methods (ERPs), perhaps such methods could instead be used for detection and/or assessment of cognitive disorders.

## Caveats for research

This literature implies that visual narratives require a fluency that develops across age and exposure, thereby challenging assumptions of their transparent understanding. These findings are problematic for researchers who use visual narratives as stimuli across several domains of the psychological sciences.

Consider the prevalent use of the PAT. Because of its inclusion in general intelligence (IQ) tests (WAIS-IQ, WISC) and clinical assessments (Kaufman & Lichtenberger, 2006; Wechsler, 1981), the PAT has been a staple of diagnosing brain damage and many other cognitive disorders. Yet, it is unclear what the PAT indexes, be it social intelligence, logical reasoning, temporal cognition, or narrative comprehension (Campbell & McCord, 1996; Ingber & Eden, 2011; Lipsitz, Dworkin, & Erlenmeyer-Kimling, 1993; Ramos & Die, 1986; Tulsky & Price, 2003). Also, such studies *never include measures of visual narrative reading experience*, despite longstanding findings that the PAT is modulated by cultural background (Breiger, 1956), and age and experience with visual narratives (A. L. Brown, 1975; Fivush & Mandler, 1985; Friedman, 1990; Nakazawa, 2016; Weist et al., 1999; Weist et al., 1997). The PAT is also confounded because it is scored relative to a “correct” order, despite multiple well-formed sequences being possible when accounting for the structure of visual narratives (Cohn, 2014b).

Issues also arise with interpreting TCATs as influenced by writing systems (Fuhrman & Boroditsky, 2010) and/or time-space metaphors (Fedden & Boroditsky, 2012). While visual narrative layouts are influenced by the direction of writing systems (Cohn, Axnér, Diercks, Yeh, & Pederson, 2019), their navigation also involves specialized fluency even without content (Cohn, 2013a). Thus, it is problematic to attribute an influence to writing or metaphors on picture arrangement if participants have exposure to visual narratives with similar or associated rules of layout as writing. No study on “temporal cognition” using a TCAT ***–*** regardless of population ***–***mentions visual narratives or participants’ familiarity with them.

In developmental research, whatever cognitive ability researchers may target may be confounded by the *concurrent development* of visual narrative fluency, which is typically not measured. For example, common assessments of theory of mind with visual narratives (Baron-Cohen et al., 1986; Sivaratnam et al., 2012) are problematic since sequential construal begins ~4 to 5 years of age, which is within or *after* the window of development for theory of mind (ToM) (Wellman et al., 2001). Without adequate measures, it is unclear whether children who fail at such tasks do so because of ToM or because of visual narrative fluency. Similar challenges face developmental research using visual narratives to assess temporal cognition (Ingber & Eden, 2011; Weist, 2009) and sequential reasoning (Zampini et al., 2017). This explains virtual reality’s advantage over PATs for assessment of children’s “temporal sequencing” (Eden & Passig, 2007), because PAT performance reflects visual narrative fluency, not temporal or causal reasoning.

Caveats also extend to narrative elicitation tasks (e.g., Berman & Slobin, 1994). Despite constituting much of the research on narrative development (Burris & Brown, 2014), with no assessment of fluency, it is unclear whether NETs index verbal, visual, or amodal narrative abilities. This may be why questioning improves children’s coherence in narrating picture stories: such questioning can verbally scaffold their basic comprehension of the visual sequences (Silva & Cain, 2017; Silva, Strasser, & Cain, 2014).

NETs also discount the structure of the visual narratives. Rarely are the properties of such materials coded, and instead are treated as structurally neutral depictions on par with real-life events. Yet, these materials *also have a narrative structure*. NETs reflect a *translation* of the narrative structure from the visual to the verbal domain, not narration elicited about conceived events alone. This may explain why verbal narrative structures are more coherent in NETs than when children tell original stories, since the visual narratives provide a structure for children to translate rather than to generate on their own (Nurss & Hough, 1985; Shapiro & Hudson, 1991). Researchers rarely analyze or acknowledge these *visual* narrative structures (cf. Berman & Slobin, 1994; Trabasso & Nickels, 1992), or the processes necessary to understand them (e.g., Karmiloff-Smith, 1985), despite them laying the foundation for all subsequent interpretations.

In sum, experimental researchers must be sensitive to confounds of using visual narratives, which include limitations of the task, sensitivity to participants’ fluency, and structural properties of the stimulus materials. This is not to admonish visual narratives in experimental tasks entirely, but their use should follow similar considerations of language-based stimuli. In linguistic tasks, researchers typically know the linguistic properties of such stimuli and participants’ fluency in that language. Similar standards should be upheld for using visual narratives.

## Conclusion

This review has explored the universality of visual narratives in comprehension and production by examining cross-cultural, developmental, and clinical contexts. To summarize:

1. Visual narratives are a fundamental and natural potential of human expression.
2. Visual narrative comprehension requires fluency acquired through exposure and practice.
3. Visual narrative fluency applies both generally, and to structures of specific systems.
4. Visual narrative fluency matures across a developmental trajectory modulated by exposure.
5. Visual narrative fluency can be asymmetrical for comprehension and production.
6. Visual narratives are complex systems, and deficits in their understanding can involve many interacting factors.
7. Visual narrative fluency may involve domain-general and cross-modal systems, but the degree to which fluency is transferable across modalities remains unclear.

These findings align with research arguing that visual narrative understanding and production is parallel to language. While the capacity to comprehend and produce images is universally accessible to any neurotypical human brain, without exposure to an external system, the requisite structures (or interfaces between structures) may not develop. Just as languages differ in systematic ways across cultures, so do visual narratives. Thus, a comprehender may have fluency in the structures in their “native visual language,” which may conflict with other systems (Cohn, 2013b; Cohn & Kutas, 2017; Nakazawa & Shwalb, 2012; Wilkins, 1997/2016).

These interpretations raise questions about advocating for sequential images under the assumption of transparency. This has practical consequences in contexts like children’s toy assembly (Martin & Smith-Jackson, 2008), instruction manuals (Spinillo & Dyson, 2001), or comics in education (Nalu, 2011; Wong, Miao, Cheng, & Yip, 2017). Similar concerns apply to visual narratives used as stimuli to test other aspects of cognition. Experimental and clinical tests using visual narratives should analyze their properties, and measure individuals’ exposure and expertise with them (e.g., VLFI, CCCT). Future research could further develop proficiency metrics and assessments for visual narrative fluency, and clarify its interactions with other cognitive systems (e.g., working memory, causal reasoning, theory of mind, etc.).

So, is visual narrative comprehension universal? The extent research suggests that they are not “universally transparent.” Rather, like language, visual narratives are “universal” in the sense that typically developing human brains innately have cognitive structures necessary to gain fluency in their understanding, given the requisite exposure to and practice with an external system across a developmental trajectory. This potentiality is a testament to their fundamental role in human expression, spanning across history and cultures. Given this, perhaps it is time that we study them with the same seriousness afforded to other basic aspects of human communication and expression.

## Footnotes

1 Resources for using the VLFI are available online at: http://www.visuallanguagelab.com/resources.html
